# An algorithm-based technique for counting mitochondria in cells using immunohistochemical staining of formalin-fixed and paraffin-embedded sections

**DOI:** 10.1007/s00432-024-05653-1

**Published:** 2024-04-03

**Authors:** Mai Sakashita, Noriko Motoi, Gaku Yamamoto, Emi Gambe, Masanori Suzuki, Yukihiro Yoshida, Shun-ichi Watanabe, Yutaka Takazawa, Kazunori Aoki, Atsushi Ochiai, Shingo Sakashita

**Affiliations:** 1 Division of Biomarker Discovery, Exploratory Oncology Research and Clinical Trial Center, National Cancer Center, Chiba, Japan; 2 Department of Pathology, Saitama Cancer Center, Saitama, Japan; 3 Division of Genome Biology, National Cancer Center Research Institute, Tokyo, Japan; 4 Division of Translational Genomics, Exploratory Oncology Research and Clinical Trial Center, National Cancer Center, Kashiwa, Japan; 5 Department of Pathology, Toranomon Hospital, Tokyo, Japan; 6 Department of Thoracic Surgery, National Cancer Center Hospital, Tokyo, Japan; 7 Department of Immune Medicine, National Cancer Center Research Institute, Tokyo, Japan; 8 Division of Pathology, Exploratory Oncology Research and Clinical Trial Center, National Cancer Center, 6-5-1, Kashiwanoha, Kashiwa-Shi, Chiba, 277-8577 Japan; 9 Research Institute for Biomedical Sciences, Tokyo University of Science, Tokyo, Japan

**Keywords:** Mitochondria, Cancer, COX4, MitoTracker

## Abstract

**Purpose:**

Visualizing mitochondria in cancer cells from human pathological specimens may improve our understanding of cancer biology. However, using immunohistochemistry to evaluate mitochondria remains difficult because almost all cells contain mitochondria and the number of mitochondria per cell may have important effects on mitochondrial function. Herein, we established an objective system (Mito-score) for evaluating mitochondria using machine-based processing of hue, saturation, and value color spaces.

**Methods:**

The Mito-score was defined as the number of COX4 (mitochondrial inner membrane) immunohistochemistry-positive pixels divided by the number of nuclei per cell. The system was validated using four lung cancer cell lines, normal tissues, and lung cancer tissues (199 cases).

**Results:**

The Mito-score correlated with MitoTracker, a fluorescent dye used to selectively label and visualize mitochondria within cells under a microscope (R^2^ = 0.68) and with the number of mitochondria counted using electron microscopy (R^2^ = 0.79). Histologically, the Mito-score of small cell carcinoma (57.25) was significantly lower than that of adenocarcinoma (147.5, p < 0.0001), squamous cell carcinoma (120.6, p = 0.0004), and large cell neuroendocrine carcinoma (111.8, p = 0.002).

**Conclusion:**

The Mito-score method enables the analysis of the mitochondrial status of human formalin-fixed paraffin-embedded specimens and may provide insights into the metabolic status of cancer.

**Supplementary Information:**

The online version contains supplementary material available at 10.1007/s00432-024-05653-1.

## Introduction

Mitochondria perform several important functions in cells, including the production of energy; generation of reactive oxygen species, redox molecules, and metabolites; regulation of cell signaling and cell death; and biosynthesis of metabolites (Zong et al. 2016; Nunnari and Suomalainen 2012). Normal cells primarily rely on mitochondrial oxidative phosphorylation for energy generation. Conversely, most cancer cells produce excess lactate in the presence of sufficient oxygen (Warburg 1956), a phenomenon known as the “Warburg effect.” Although Warburg originally considered this effect to reflect mitochondrial dysfunction in cancer cells, many cancer cells exhibiting the Warburg effect may possess intact mitochondrial function but altered metabolism (Vander Heiden et al. 2009). Mutations in mitochondrial DNA contribute to tumor progression by enhancing the metastatic potential of cancer cells (Ishikawa et al. 2008). In small cell lung cancer, pyruvate kinase M1 converts phosphoenolpyruvate to pyruvate during glycolysis and promotes tumor growth (Morita et al. 2018). In lung adenocarcinoma, the ovarian cancer immunoreactive antigen domain-containing 2, which may cause malignant transformation in lung adenocarcinoma at an early phase, may be a key factor in maintaining mitochondrial function or structure (Sakashita et al. 2018). The ability to observe mitochondria in cancer cells from human pathological specimens may improve our understanding of the relationship between mitochondrial status and cancer. Although many studies have explored mitochondria in various contexts, relatively few have examined mitochondria in human cancer histological specimens using immunohistochemistry (IHC) primarily because this method has several limitations. For instance, almost all cells contain mitochondria; therefore, determining a cutoff value for positive IHC staining is impossible. The enumeration of mitochondria is necessary to determine their functions; however, counting the number of mitochondria using IHC remains challenging. The quantity of mitochondria may also be correlated with cellular functions, such as metabolism; however, this correlation in each cell is difficult to establish using light microscopy. Recently, digital pathology has been demonstrated to have a relatively high accuracy for the automated detection of IHC samples, thereby eliminating subjectivity (Harmon et al. 2021; Wetstein et al. 2021). Herein, we developed an objective evaluation system based on the hue, saturation, and value (HSV) color space to determine the immunohistochemical status of mitochondria using formalin-fixed and paraffin-embedded (FFPE) sections. This system enabled separation of the color of the IHC (DAB) from that of the stained nucleus (hematoxylin); thus, we simultaneously measured the IHC score and number of nuclei. This system was validated using four lung cancer cell lines and FFPE sections obtained from normal tissues. This system was also used to evaluate the status of mitochondria in resected human lung cancer specimens.

## Materials and methods

### MitoTracker analysis using lung cancer cell lines

A549, NCI_H358, NCI_H1373, and NCI_H1975 cells were seeded at 1.5 × 10^5^ cells/well in 6-well plates the day before the experiment. MitoTracker™ RED FM (M22425) was added at a concentration of 100 nM and incubated for 30 min at 37 °C in a humidified incubator with 5% CO_2_. Cells were then harvested and analyzed via fluorescence cytometry using a BD FACSCanto IITM (Becton Dickinson), and the mean fluorescence intensity was calculated using FlowJoTM software (ver 10.7.1).

### Electron microscope analysis using lung cancer cell lines

For electron microscopic analysis, cells were fixed with 2.5% glutaraldehyde (pH 7.2, 0.1 M Phosphate buffer) and then cell blocks were prepared. After creating the cell blocks, Epon embedding was performed, and after ultrathin sectioning, analysis was performed using an electron microscope (JEM-1400Flash, JEOL Tokyo, Japan). Then, 10 cells with the largest split surfaces were selected, and the number of mitochondria was visually measured and subsequently averaged.

### Tissues samples

Lung carcinoma specimens were used that had been surgically resected from patients originally diagnosed at the National Cancer Center Hospital (Tokyo, Japan) between 2017 and 2019. Tumor slides and blocks of samples from 199 patients were used for histological evaluation and tissue microarray (TMA) construction. The clinicopathological characteristics of the patients are summarized in Table 1. To evaluate the validity of the system, we also used specimens containing only normal tissues from patients with surgically resected stomach and kidney carcinomas at the National Cancer Center Hospital (Chiba, Japan) in 2021. The clinical data of all patients were collected from their medical records. Informed consent was obtained from all patients for the use of their materials. The Institutional Review Board approved the use of the specimens (2016-124, National Cancer Center, Japan, 2020-242, National Cancer Center East, Japan). The study was conducted in accordance with the principles of the Declaration of Helsinki.

**Table 1:** Patient clinicopathological information

		Total	AD	SQ	Small	LCNEC
Case number		199	96	57	27	19
Gender	Female	67	45	9	8	5
	Male	132	51	48	19	14
Age	Average	68	66	71	69	69
pT factor	T1	52	31	8	11	2
	T2	74	29	22	14	9
	T3	52	30	15	2	5
	T4	21	6	12	0	3
pN factor	N0	126	57	38	18	13
	N1	30	11	10	6	3
	N2	42	27	9	3	3
	N3	1	1			
pM factor	M0	192	90	56	27	19
	M1	7	6	1	0	0
pStage	I	79	40	14	17	8
	II	54	18	23	7	6
	III	59	32	19	3	5
	IV	7	6	1		
Subtype						
Adenocarcinoma	Lepidic		3			
	Acinar		11			
	Papillary		37			
	Solid		23			
	Micropapillary		15			
	IMA		4			
	Other (enteric, colloid and fetal)		4			
Squamous cell carcinoma	W/D			7		
	M/D			38		
	P/D			12		

*AD* adenocarcinoma, *SQ* squamous cell carcinoma, *Small* small cell carcinoma, *LCNEC* large cell neuroendocrine carcinoma

### IHC using TMAs and cell blocks of the lung cancer cell lines

TMAs, comprising 2-mm cores with two cores from each tumor, were constructed from 10% FFPE blocks. The two cores were separately embedded in two blocks. Four representative tumor areas were marked on hematoxylin and eosin-stained slides. In total, 796 specimens had adequate cores for immunohistochemical analysis.

For cell blocks of the lung carcinoma cell lines, cells were sedimented by centrifugation, fixed in 10% neutral buffered formalin, embedded in paraffin blocks, and thinly sliced to 4 μm.

IHC was performed on 3-µm–thick sections prepared from TMAs. Sections were deparaffinized and rehydrated, and antigen retrieval was performed using Cell Conditioning solution, CC1 (EDTA, pH 8.5; Roche, Basel, Switzerland) at 95 °C for 64 min. Immunohistochemical staining was performed using rabbit polyclonal anti-cytochrome c oxidase subunit IV isoform 1 antibody diluted at 1:500 (HPA002485, Sigma-Aldrich, St. Louis, MO, USA) for 1 h at room temperature using a Roche Ventana BenchMark Ultra autostainer and Ventana Ultra View Universal DAB Detection kit (Roche), and using rabbit polyclonal antivoltage-dependent anion channel 1 (VDAC1) antibody diluted at 1:50 (HPA030780, Sigma-Aldrich, St. Louis, MO, USA) for 1 h at room temperature using a Roche Ventana BenchMark Ultra autostainer and Ventana Ultra View Universal DAB.

An antibody specific for cytochrome c oxidase subunit IV isoform 1, a marker of the mitochondrial inner membrane, was selected because the granular staining pattern in the cytoplasm clearly indicates the presence of mitochondria.

### IHC evaluation using whole slide images

Stained TMA were scanned at 40× magnification using a whole slide scanner NanoZoomer S360 (Hamamatsu Photonics, Hamamatsu, Japan). A pathologist annotated two circled areas of the carcinoma and stroma (0.08 m^2^ each) using Labelme software (Wada 2016). We preferentially selected areas where the carcinoma and stroma were physically separated because accurately annotating overlapping areas of carcinoma and stroma was difficult as these areas include inflammatory cells, macrophages, and vascular endothelium.

**Fig. 1:**
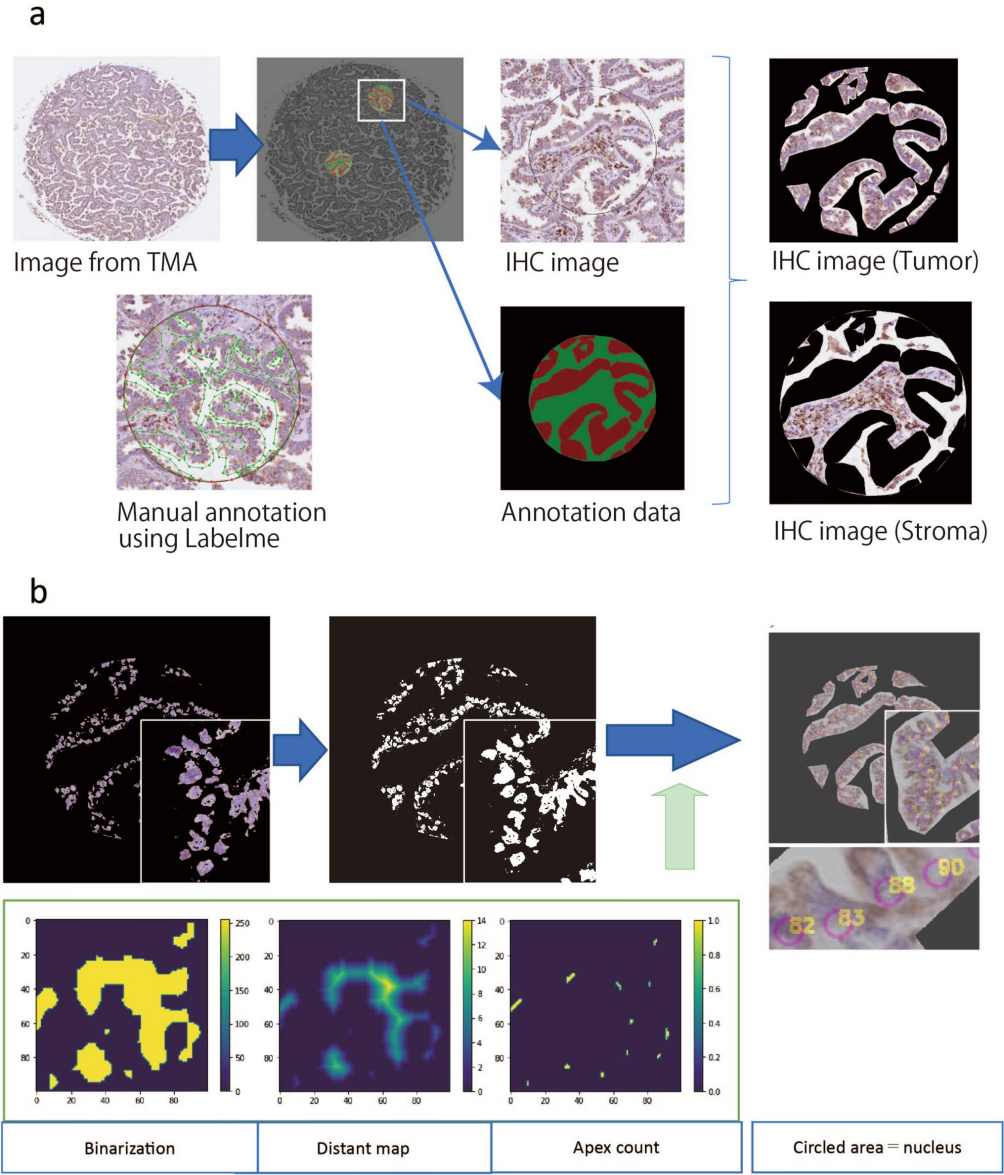
IHC scoring and nucleus counting using a machine-based system. **a** Overview of manual separation of carcinoma and stroma from tissue microarray. *TMA* tissue microarray, *IHC* immunohistochemistry/immunohistochemical. **b** Nuclear counting system. Image thresholding of the nucleus was performed using Otsu’s thresholding technique (Otsu 1979). After denoising, a distance map was created by calculating the distances between the background and nucleus. Finally, apexes of the distance map were counted to determine the number of nuclei using digital image processing. The apexes were circled by a computer for validation as nuclei by certified pathologists

A pixel-based approach for the spatial annotation of mitochondrial expression was used. The image processing steps are illustrated in Fig. 1a. All image patches were derived with reference to 2000 × 2000-pixel regions under a 40× objective within the annotated regions of the carcinoma.

### Extraction of IHC staining and nucleus by converting an acquired red, green, and blue image to HSV color space

We transformed the image from the red, green, and blue color spaces into the HSV color space using the OpenCV (3.4.15) library. Cells were then separated from the background by changing the value (V) component and applying V to eliminate the background. A V value of 10–220 was adopted for nuclear separation and 0–160 was used for IHC separation.

**Fig. 2:**
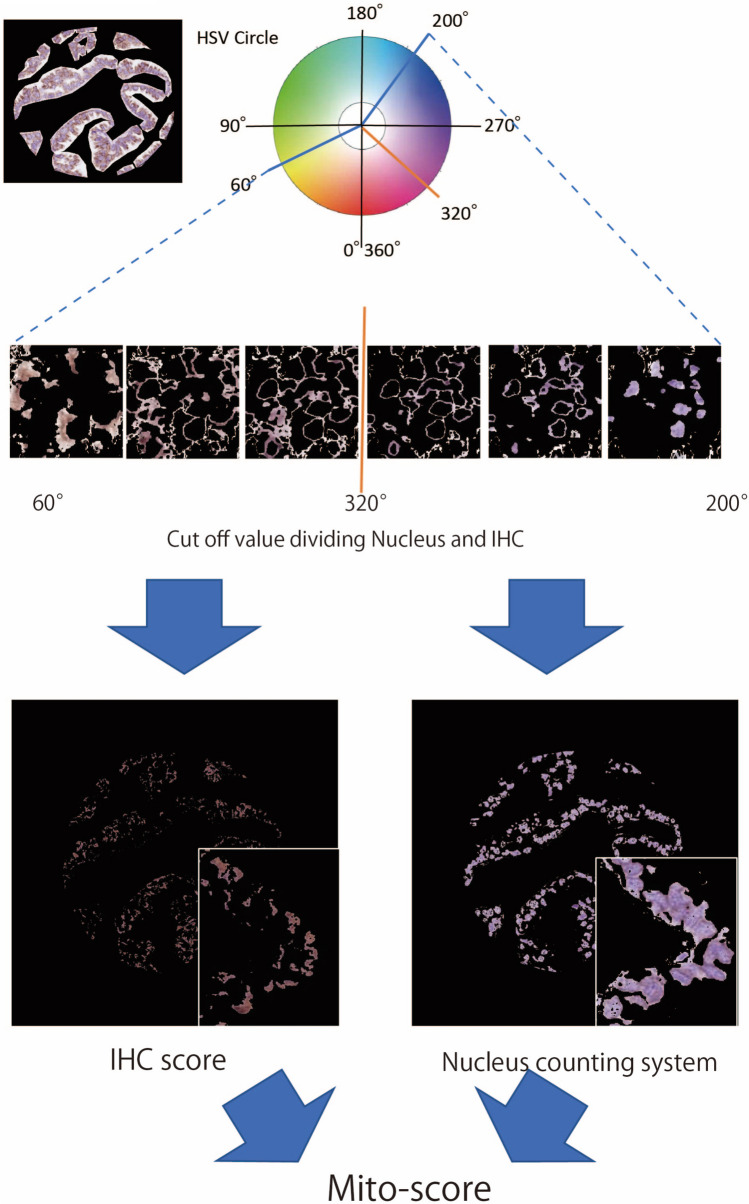
Extraction of nucleus and immunohistochemistry color results using the hue component. Hues of 0°–60° and 320°–360° were determined as the color of the immunohistochemistry results, and hues of 200°–320° were determined as the color of the nuclei. *HSV* hue, saturation, value, *IHC* immunohistochemistry

We extracted the color of the IHC (brownish color of DAB) and nuclei (bluish color of hematoxylin) using the hue (H) component. H values of 0°–60° and 320°–360° were determined as the color of IHC staining, and H values of 200°–320° were determined as the color of the nucleus by certified pathologists (Fig. 2).

Using this process, we classified the pixels according to whether they were annotated as cells (carcinoma or stroma) or background. Nonspecific staining was eliminated using the saturation (S) component. An S value of 20–255 was used to separate the nuclei while a value of 60–255 was used to separate the IHC staining.

### Evaluation of mitochondria IHC

To evaluate mitochondria, the Mito-score was defined as the number of COX4 IHC-positive pixels divided by the number of nuclei, as we hypothesized that the number of mitochondria per cell was related to their function. COX4 IHC-positive pixels were counted after separation by IHC staining. The nuclear counting system was as follows (Fig. 1b). First, the image threshold of the nucleus was obtained using Otsu’s thresholding technique or discriminant analysis (Otsu 1979). Rough cell edges and holes in the nucleus can cause an inaccurate nuclear count. Therefore, we performed the following steps to reduce the image noise (denoising). Dilation was conducted to smooth the nucleus edges by gradually enlarging the boundaries of foreground pixel regions. Closing was performed to remove the holes in the nucleus. Next, a distance map was created by calculating the distance between the background and the nucleus. Erosion was used to remove the boundaries of the foreground pixel regions. Overlapping cells can be counted as single cells, causing inaccurate cell counting. The apex of the nucleus was extracted and magnified to reduce any inaccuracy. The position of the center of gravity was calculated using the number, position, and contour of the nucleus. Nuclei were enumerated using digital image processing and the apex was circled using a computer. Certified pathologists confirmed the circled area as the nucleus. However, this counting system may not be effective for crowded cells. In such cases, the denoising treatment was changed and the calculations were repeated. Finally, the Mito-score was calculated as the number of COX4 IHC-positive pixels divided by the number of nuclei.

### Statistical analysis

Statistical analyses were performed using GraphPad Prism software (version 9; GraphPad Inc., La Jolla, CA, USA). Significance was defined as p < 0.05. The association between Mito-score and various subtypes of lung carcinoma was analyzed using an unpaired *t*-test. The correlation between the Mito-scores of the tumor and stroma was assessed using linear regression analysis.

## Results

### Method validation using lung cancer cell lines

**Fig. 3:**
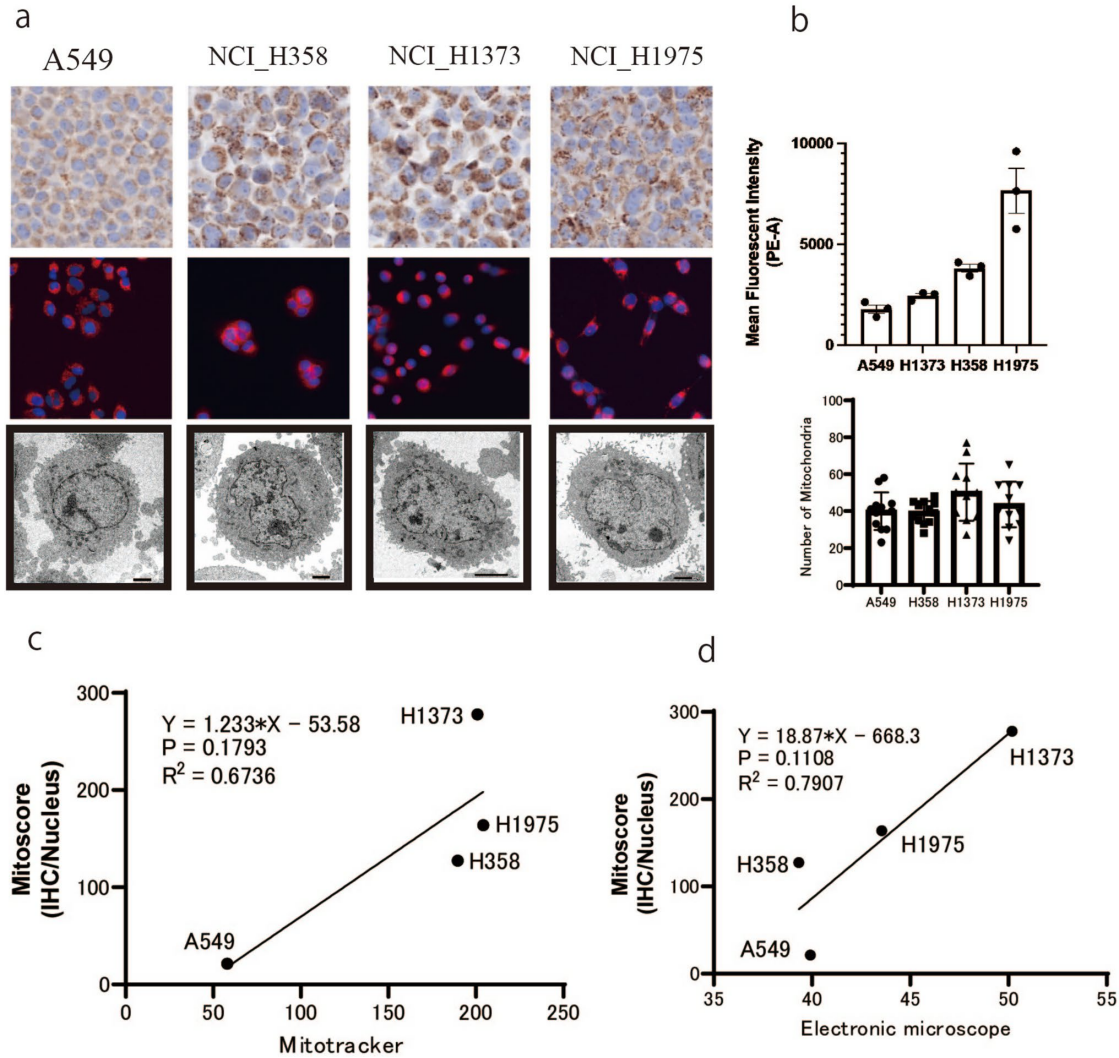
Method validation using four lung cancer cell lines. **a** COX IV (IHC), MitoTracker, and electron microscopy images for each lung cancer cell line. **b** Distribution of MitoTracker fluorescence intensity and number of mitochondria for each cell lines. **c** Correlation analysis between MitoTracker and Mito-score. **d** Correlation analysis between electronic microscope and Mito-score

Four lung cancer cell lines (A549, NCI_H358, NCI_H1373, and NCI_H1975) were used to identify correlations between general mitochondrial abundance measurements and the mitochondrial abundance measurements obtained using Mito-score. Specifically, we measured the correlation between measurements by MitoTracker and Mito-score (Fig. 3a–c), and between actual measurements by electron microscopy and Mito-score (Fig. 3a, b, d).

In the MitoTracker measurement, the correlation analysis had an R^2^ = 0.6736, suggesting a correlation between the MitoTracker and Mito-score (Fig. 3c). Correlation analysis also showed a correlation of R^2^ = 0.7907 with the actual values measured by electron microscopy (Fig. 3D). These results indicate that the proposed method correlates with existing methods for measuring mitochondrial abundance and that this can be measure the number of mitochondria can be achieved using IHC.

### Validation of evaluation method using single cells

**Fig. 4:**
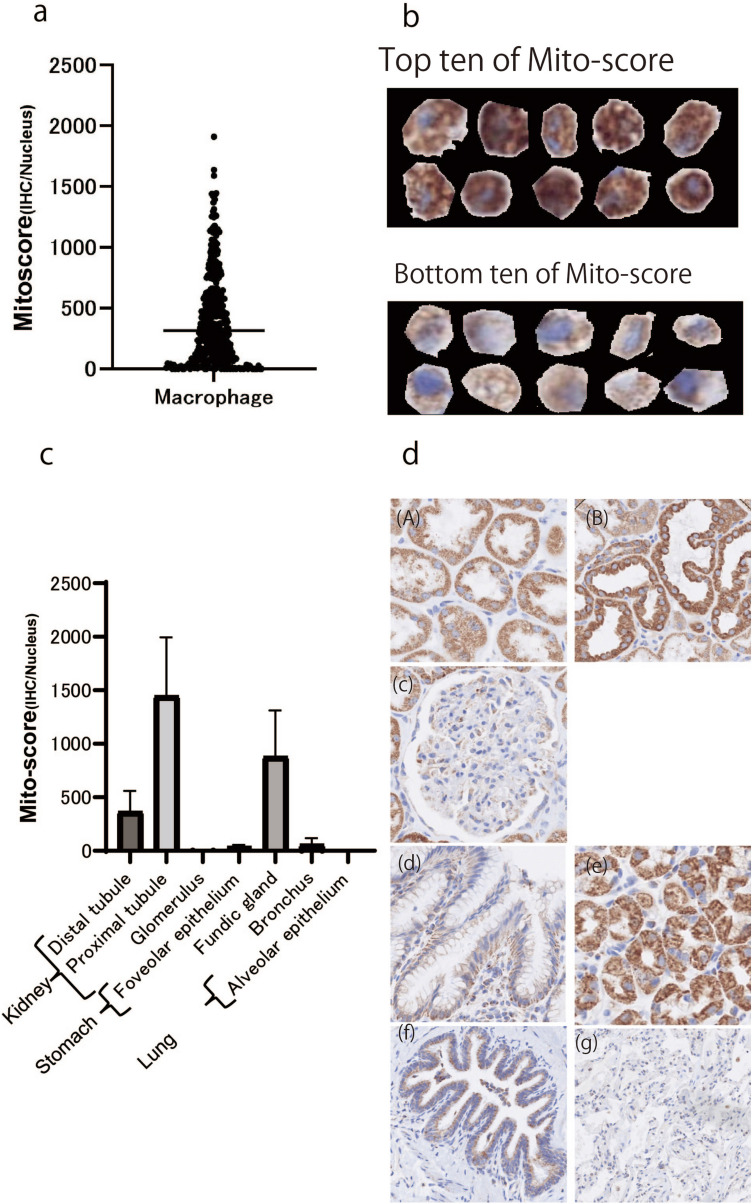
Validation of the method using normal tissue. **a** Validation of the evaluation method using macrophages as single cells. Macrophages showed variable Mito-scores. **b** Mito-score of macrophages. Images of the top ten and bottom 10 Mito-scores are shown. **c** Mito-score of normal kidney, stomach, and lung tissues. **D** COX4 IHC image of normal tissue. **a** Distal tubule, **b** proximal tubule, **c** glomerulus, **d** foveolar epithelium, **e** fundic gland, **f** bronchus, and **g** alveolar epithelium

The evaluation method was validated using a single-cell model. We selected macrophages for validation because macrophage boundaries do not make contact with each other, and each single cell can be easily annotated. Macrophages showed variable mitochondrial staining patterns (Fig. 4a), and their Mito-scores were calculated. Images with the top and bottom 10 Mito-scores are shown in Fig. 4b.

### Validation of evaluation method using normal tissue

**Fig. 5:**
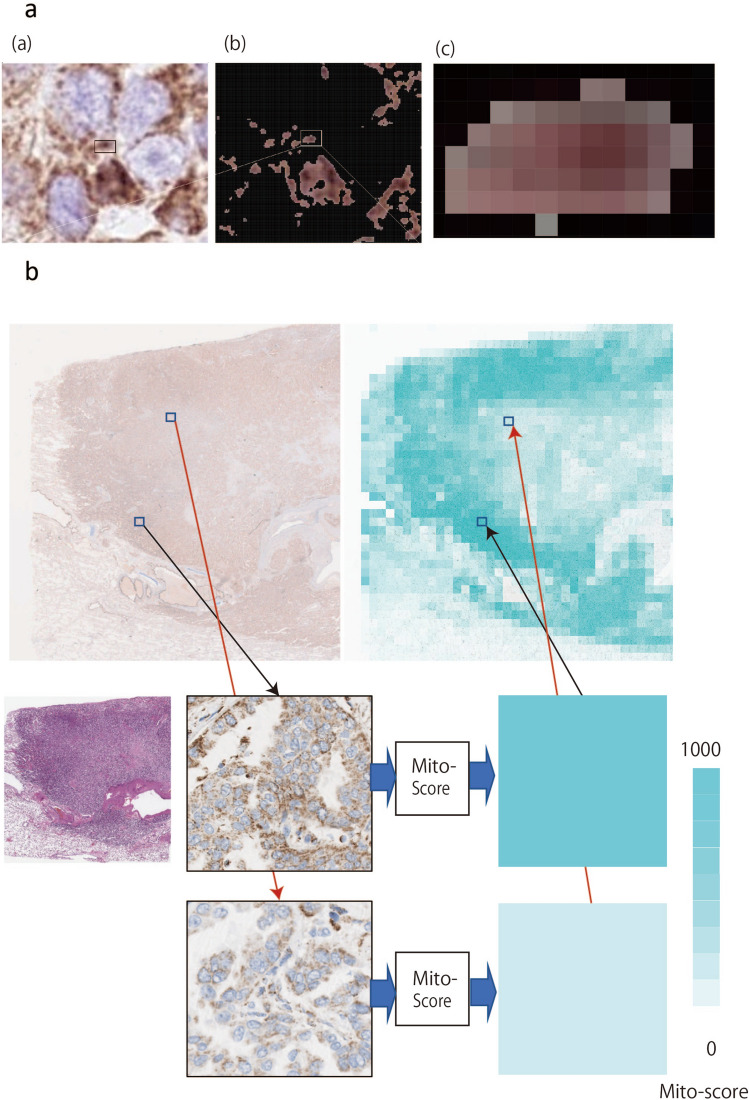
IHC and digital image. **A** COX4 IHC image and extracted COX4 IHC image using HSV color space. **a** COX4 IHC images are shown. **b**, **c** Extracted COX4 IHC images using the HSV color space. **c** Magnified image of **a** (**b**). **b** Mito-score of lung adenocarcinoma using whole section. The Mito-score of adenocarcinoma was obtained from the COX4 IHC image using our system. The objective numerical number was obtained using the Mito-score, and the mitochondria distribution in lung adenocarcinoma is shown in a heatmap

The evaluation method was validated using normal tissues obtained from surgically resected kidney, stomach, and lung carcinomas without cancer cells. The Mito-scores are shown in Fig. 4c, and COX4 IHC images of normal tissues are shown in Fig. 4d. In the kidneys, the average Mito-scores for the proximal tubule, distal tubule, and glomerulus were 1446, 365, and 5.7, respectively. In the stomach, the average Mito-scores for the fundic gland and foveolar epithelium were 880 and 38, respectively. In the lungs, the average Mito-scores for the bronchus and alveolar epithelium were 58.5 and 0.00031, respectively. Next, we used digital imaging to extract each COX4-stained granule. Some granules were in the range of 1–2 µm in size, which is the reported size range for mitochondria (Fig. 5a) (Egner et al. 2002); moreover, in this image, one pixel was 0.23 µm; thus, the resolution was sufficient to reflect the size of mitochondria.

In the kidneys, the proximal and distal tubules contain many mitochondria because active transport is required for ion absorption. The proximal tubules contain more mitochondria than any other structure in the kidneys because higher levels of active transport are required in this region (Bhargava and Schnellmann 2017). Conversely, glomerular filtration is a passive process, and the glomeruli do not require large amounts of energy. In the stomach, parietal cells contain many mitochondria (Lawn 1960). The proximal tubule and fundic gland showed relatively high Mito-scores, which is consistent with the results of previous studies validating the proposed system (Bhargava and Schnellmann 2017).

### Evaluation of mitochondria in lung carcinoma and stroma

Our system was used to evaluate the surgically resected lung carcinomas. Using whole sections of lung adenocarcinoma sections (Fig. 5b), the Mito-score was obtained from the COX4 IHC images. The objective numerical value was determined from the Mito-score, and the mitochondrial distribution was observed in a heatmap.

**Fig. 6:**
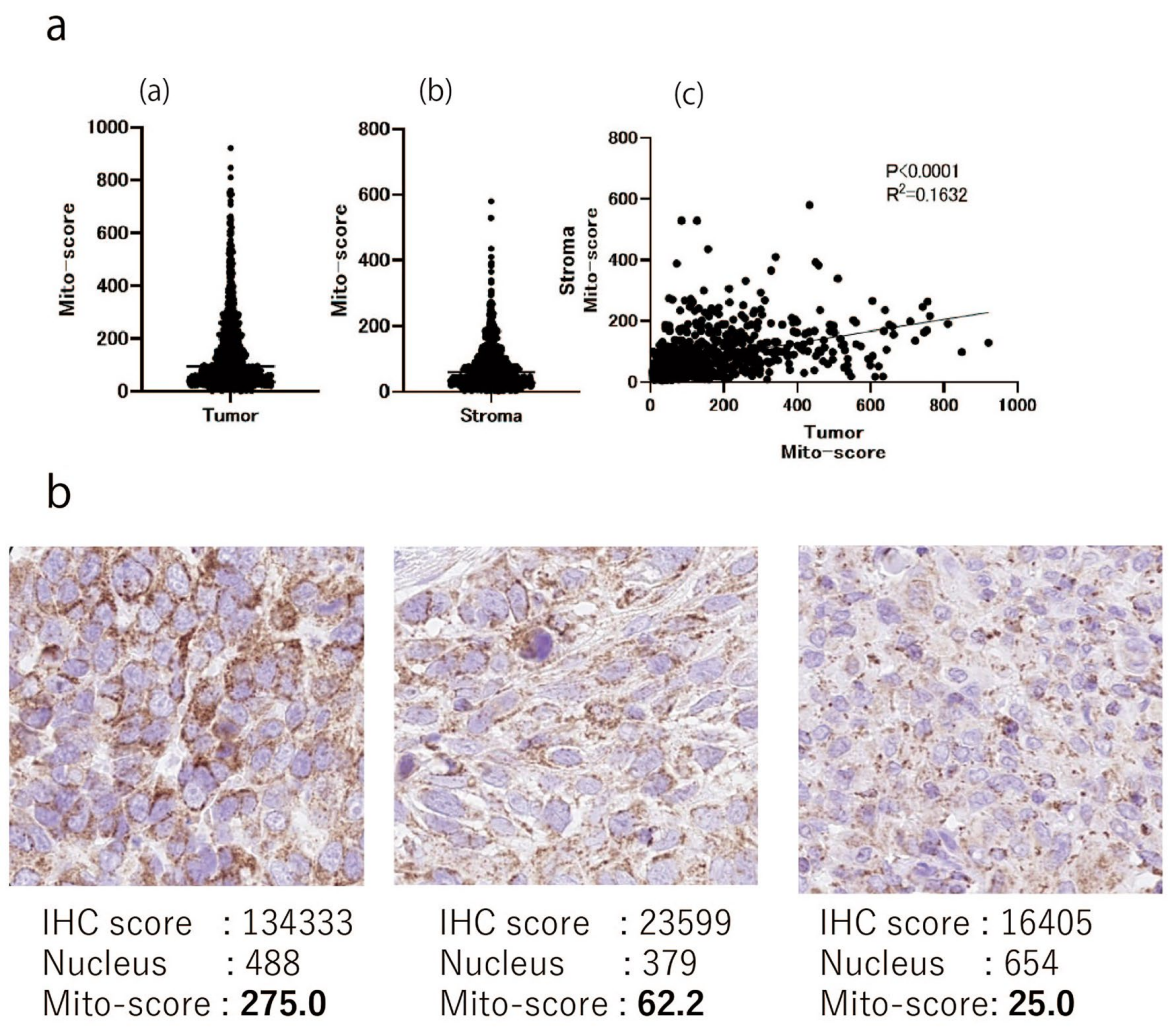
Mito-score of lung carcinoma. **A** Mito-score of tumor and stroma. **a** Mito-score of tumor. **b** Mito-score of stroma. **c** Correlation of Mito-score of tumor with Mito-score of stroma. **B** Representative image of COX4 IHC staining of lung carcinoma. The IHC score, nucleus count, and Mito-score are shown

The Mito-score of the lung carcinoma and stroma varied from high to low (Fig. 6a (a, b)). The Mito-score of the stroma varied because of the presence of macrophages, lymphocytes, plasmacytes, fibroblasts, and the vascular endothelium. The Mito-score of the tumor showed a weak positive correlation with that of the stroma (R^2^ = 0.1632, p < 0.0001) (Fig. 6a (c)). To evaluate the mitochondrial score of the carcinoma, the stroma was excluded from the mitochondrial evaluation using annotation software, and representative images of the top, middle, and bottom Mito-scores for lung carcinoma are shown in Fig. 6b.

### Calculation of Mito-score in various histological subtypes of lung carcinoma

**Fig. 7:**
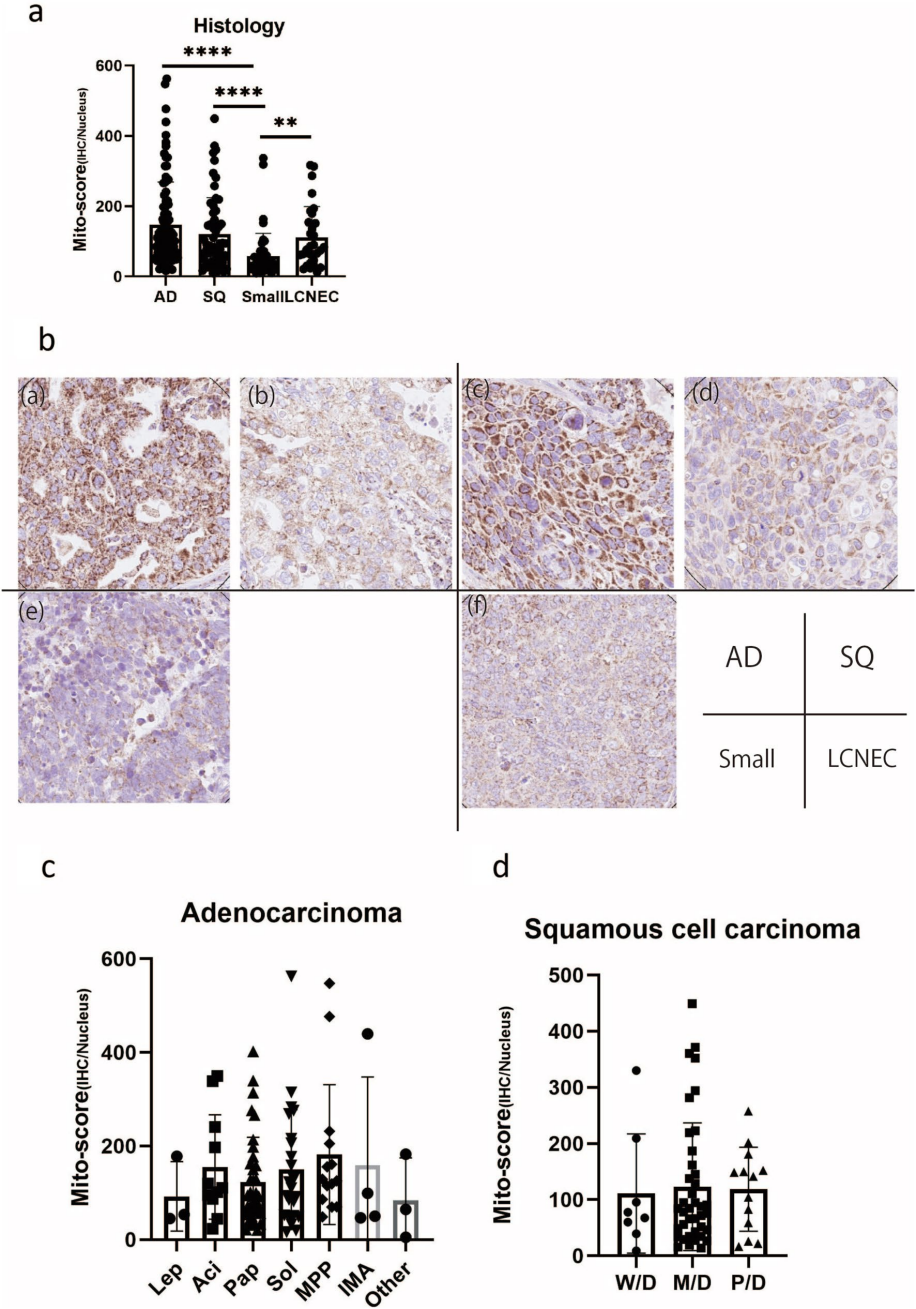
Mito-score of various subtypes of lung carcinoma. **A** Mito-scores of various subtypes of lung carcinoma, including adenocarcinoma (AD), squamous cell carcinoma (SQ), small cell carcinoma (Small), and large cell neuroendocrine carcinoma (LCNEC) were examined. The Mito-score of small cell carcinoma (57.25) was significantly lower than that of AD (147.5, p < 0.0001), SQ (120.6, p = 0.0004), and LCNEC (111.8, p = 0.002). **b** Representative COX4 IHC images of several subtypes of lung carcinoma. **c** Mito-score of AD. In AD, no significant differences were observed among lepidic, acinar, papillary, solid, or micropapillary subtypes. **d** Mito-score of SQ. In SQ, no significant difference was observed among differentiation status: well differentiated (W/D), moderately differentiated (M/D), and poorly differentiated (P/D)

The Mito-scores of various subtypes of lung carcinoma, including adenocarcinoma, squamous cell, small cell, and large cell neuroendocrine carcinoma, were examined (Fig. 7a). The COX4 IHC-stained images of these carcinomas are shown in Fig. 7b. The Mito-score of small cell carcinoma (57.25) was significantly lower than that of adenocarcinoma (147.5, p < 0.0001), squamous cell carcinoma (120.6, p = 0.0004), and large cell neuroendocrine carcinoma (111.8, p = 0.002). The Mito-score of adenocarcinoma did not significantly differ from that of squamous cell carcinoma (p = 0.16). Adenocarcinomas and squamous cell carcinomas were further analyzed according to their subtypes. In adenocarcinomas, no significant differences were observed between the lepidic, acinar, papillary, solid, and micropapillary subtypes (Fig. 7C). In squamous cell carcinoma, no significant differences were observed among various degrees of differentiation (well, moderately, and poorly differentiated) (Fig. 7D).

## Discussion

Herein, we established an objective mitochondrial evaluation system using machine-based processing based on the HSV color space obtained using human FFPE samples. Except for studies that have used cultured cells for automated mitochondrial analysis (live cell imaging) (Zahedi et al. 2018), few reports have described the immunohistochemical evaluation of mitochondria in human specimens. We selected an antibody specific for COX4, a marker of the mitochondrial inner membrane, for IHC analysis to evaluate the mitochondria. We also examined the expression of voltage-dependent anion-selective channel protein 1 (VDAC1), a mitochondrial outer membrane marker. The granular staining pattern of the cytoplasm was better distinguished using COX4 as a marker than VDAC1 (Figure S1), and we therefore selected COX4 to evaluate the mitochondria using digital image processing.

The granular staining pattern of mitochondria is difficult to evaluate using conventional methods, such as the H-score (Budwit-Novotny et al. 1986). The H-score was evaluated from the area of carcinoma, which was defined as the summed percentage of positively stained cells multiplied by the weighted intensity of staining (Budwit-Novotny et al. 1986). Therefore, we could not count the number of positive cells per nucleus using the H-score because this can be biased by the density of cancer cells and the types of coexisting cells.

Pathologists typically recognize the color of IHC-prepared samples as dark, light, or slightly light brown, and this perception can be expressed in the HSV color space. In our system, the hue component corresponds to all colors and describes the most dominant wavelength. The bluish color of the nucleus (hematoxylin) and dark brownish color of the mitochondria (DAB) were objectively expressed using the hue color component. The value (V) component of HSV, which corresponds to brightness, was used to remove the background. Saturation is a basic measure of the purity or gray level of a pixel. Thus, the granular staining pattern of mitochondria can be distinguished from that of other cells based on their hue properties (Wang et al. 2021), with the HSV color model providing additional information on color, shade, and brightness (Shete and Kharate 2015; Yabusaki et al. 2014). Therefore, we used the HSV color space to evaluate IHC staining and observed that the granular staining pattern of the mitochondria was well distinguished from nuclear staining.

We hypothesized that mitochondrial function could be predicted by determining the number of mitochondria per cell. Therefore, to evaluate the mitochondria, we defined the Mito-score as the number of COX 4 IHC-positive pixels divided by the number of nuclei. We validated the method by comparing this with the conventional method, MitoTracker, and electron microscopy using lung cancer cell lines, and a correlation with the Mito-Score method was obtained. This method was validated using a single cell to examine whether the score calculated by this system correlated with the conventional visual examination and obtained consistent results. The system was validated using normal tissues such as proximal tubules from the kidneys and fundic glands of the stomach (Bhargava and Schnellmann 2017; Lawn 1960). Thus, this system can be used to evaluate various carcinomas, including lung carcinomas. Digitation offers several advantages. The number of mitochondria per cell can be quantified, and the distribution of the Mito-score can be visualized for an entire section by converting the score into a heatmap. In addition, the mitochondria of the tumors and stroma can be analyzed separately by adding annotations. Finally, several histological subtypes were analyzed. Mitochondrial quantification revealed heterogeneity in mitochondrial distribution, which was more apparent across the entire section (Fig. 4b).

Interestingly, the Mito-score of small cell carcinoma was significantly lower than that of the other subtypes of lung carcinoma. Small cell carcinoma, which has a high mitotic count, has a very poor prognosis (2-year survival rate of approximately 8%) (Nicholson et al. 2016). Both glucose and nitrogen metabolism are altered during the malignant progression of carcinoma (Kodama et al. 2020; Zhang et al. 2017), and elevated levels of c-Myc induce the expression of enzymes, such as phosphoribosyl pyrophosphate aminotransferase (PPAT), which is involved in nucleotide biosynthetic pathways (Cunningham et al. 2014; Liu et al. 2008). The fate of glutamine nitrogen is steered from the anaplerotic pathway to the tricarboxylic acid cycle in the mitochondria to enhance nucleotide biosynthesis (Kodama et al. 2020). This shift is controlled by PPAT, and the induced expression of this enzyme enhances nucleotide biosynthesis. PPAT expression is particularly strong in small cell lung carcinoma (Kodama et al. 2020); thus, the tricarboxylic acid cycle is inhibited in the mitochondria, which may explain the low Mito-score. However, further analysis is required to confirm this hypothesis.

The Mito-scores of adenocarcinoma and squamous cell carcinoma varied, and no significant difference was observed among the subtypes of these carcinomas (Fig. 6). Therefore, the number of mitochondria did not correlate with subtype of these cancers.

Although our technique is expected to have wider application in clinical settings, this study has several limitations. Firstly, a key limitation of our method is its reliance on the staining conditions of the specific facility, which may limit its applicability to other datasets. Unlike methods based on Deep Learning, our approach allows for the adjustment of parameters to tailor the conditions to each facility, suggesting a potential for tuning to suit different settings. However, this aspect has not been thoroughly investigated. Similarly, while Machine Learning or Deep Learning techniques have been widely used for nuclear identification, these AI-based methods are also susceptible to variations in staining conditions across different facilities, which may pose challenges in achieving consistent performance. The adaptability of our method in comparison to these AI-based techniques, particularly in the context of differing staining conditions, remains unexplored, as our study does not include a comparative analysis with these methods. Second, mitochondria are dynamic organelles and their morphology and other factors change in response to external stimuli and metabolic cues (Wai and Langer 2016). We hypothesized that the number of mitochondria reflects their function; therefore, we analyzed the number of mitochondria. However, whether mitochondrial number reflects functional status requires further confirmation. Third, we did not analyze the mutation status of mitochondrial DNA in carcinoma or the correlation between the number of mitochondria and the metabolic state. Among the subtypes, the Mito-scores of adenocarcinoma and squamous cell carcinoma varied, and several carcinomas showed markedly high or low Mito-scores that could not be explained by the histological type. Further studies are needed to overcome these limitations.

In conclusion, we established an objective machine-based evaluation system for enumerating mitochondria based on the IHC analysis of FFPE sections. This system enables the measurement of a value reflecting the number of mitochondria per cell (Mito-score) and the visualization of the distribution of mitochondria. The Mito-score of small cell lung carcinoma was significantly lower than that of other subtypes of lung carcinoma. This objective evaluation system can be used for the detailed examination of mitochondria in human FFPE specimens.

## Supplementary Information

Below is the link to the electronic supplementary material.

Supplementary file 1—Figure S1. Comparison between COX4 and VDAC1. Granular staining pattern of the cytoplasm was better distinguished with COX4 as a marker than with VDAC1 (JPG 1663 KB)

## Data Availability

The data that support the findings of this study are available from the corresponding author [Shingo Sakashita] upon reasonable request.
