# Supplementary figures and images for: An algorithm-based technique for counting mitochondria in cells using immunohistochemical staining of formalin-fixed and paraffin-embedded sections
Source: J Cancer Res Clin Oncol. 2024 Apr 3;150(4):172. doi: 10.1007/s00432-024-05653-1 (PMC10987345; doi:10.1007/s00432-024-05653-1)

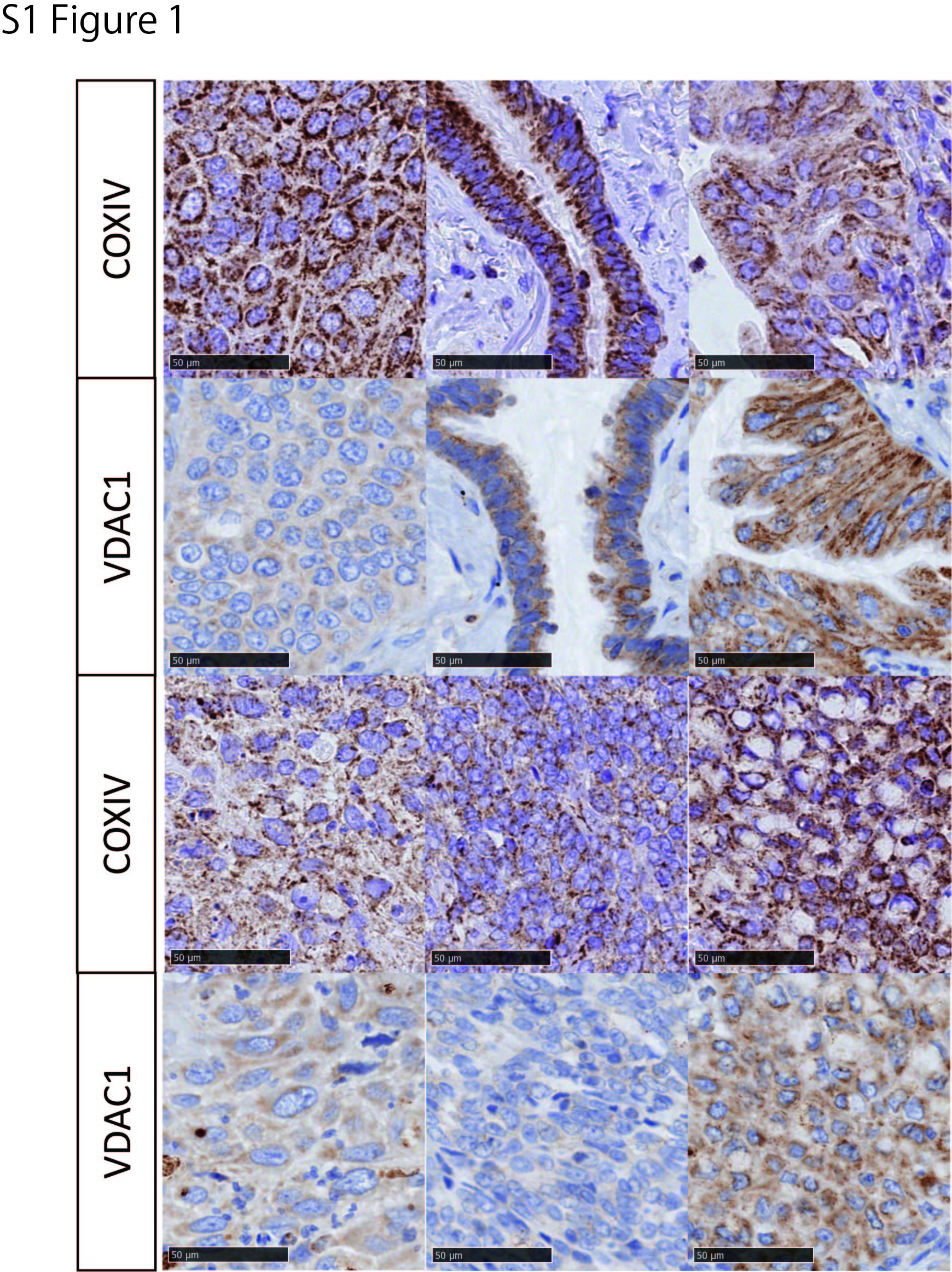

Supplement: Supplementary file 1 — Figure S1. Comparison between COX4 and VDAC1. Granular staining pattern of the cytoplasm was better distinguished with COX4 as a marker than with VDAC1 (JPG 1663 KB) [file 432_2024_5653_MOESM1_ESM.jpg]
